# The economic impact of violence perpetration in severe mental illness: a retrospective, prevalence-based analysis in England and Wales

**DOI:** 10.1016/S2468-2667(19)30245-2

**Published:** 2020-02

**Authors:** Morwenna Senior, Seena Fazel, Apostolos Tsiachristas

**Affiliations:** Department of Psychiatry, University of Oxford, Oxford, UK; Department of Psychiatry, University of Oxford, Oxford, UK; Health Economics Research Centre, Nuffield Department of Population Health, University of Oxford, Oxford, UK

## Abstract

**Background:**

Calls for increased funding for mental health services require many lines of evidence in support, including estimates of economic impact. One understudied source of cost is violence perpetrated by individuals with severe mental illness. Estimating this economic impact can inform budget planning across several government sectors and emphasise the importance of violence prevention. Therefore, we aimed to provide a comprehensive estimate of the economic costs of violence perpetrated by people with severe mental illness.

**Methods:**

For this retrospective analysis, we used a prevalence-based modelling approach to estimate the annual economic cost of violent incidents committed by people with severe mental illness in England and Wales during 2015–16. The model was based on secondary data, including the association between violence and severe mental illness, illness prevalence, recidivism, absolute numbers of violent incidents in 2015–16, and costs to society per violent crime, by area of spending. Uncertainty was addressed with probabilistic and deterministic sensitivity analyses that tested the effect of underreporting of domestic violence and distributions of crime types in individuals with severe mental illness.

**Outcomes:**

The estimated annual economic impact of violence perpetrated by people with severe mental illness was *£*2·5 (95% CI 1·4–4·5) billion in England and Wales in 2015–16, or 5·3% of the total estimated societal cost of violence. The largest contributors to the cost of violent crime perpetrated by individuals with severe mental illness were the cost of physical and emotional harm to victims (*£*1·4 [95% CI 0·8–2·5] billion), followed by lost productivity of victims (*£*348.0 [190·0–628·8] million), while the combined cost to the police and criminal justice system was *£*561·3 (305·9–1009·2) million and the cost to health services was *£*136·7 [74·3–246·3] million. The additional cost to secure forensic care was estimated to be *£*487·7 (302·0–709·1) million.

**Interpretation:**

The economic impact of violence perpetrated by individuals with severe mental illness is potentially important. Preventing violence, especially through services for individuals with comorbid substance misuse, and reducing recidivism might lead to cost savings at a governmental and individual level, in addition to the clinical and societal benefits.

**Funding:**

Wellcome Trust, National Institute for Health Research (NIHR) Oxford Biomedical Research Centre, and NIHR Applied Research Collaboration Oxford and Thames Valley.

## Introduction

Violence perpetration is a rare, but important, negative outcome for individuals with severe mental illness. The consequences extend to victims and perpetrators, who might face restricted liberty, stigma, and disrupted personal and therapeutic relationships. A public health approach to violence has been advocated because of its substantial contribution to mortality and morbidity worldwide,^1^ its large economic burden,^2^ and the societal importance of crime prevention. Such prevention has potential to reduce stigma and substantial harm. At the same time, the wider context of high rates of victimisation in people with severe mental illness needs consideration. One UK investigation, published in 2015, reported a 5-times increase in rates of victimisation in individuals with severe mental illness;^3^ victimisation, in turn, can trigger violence perpetration.^4^ Additionally, the contribution of substance misuse comorbidity has been estimated to double the risk of violent crime perpetration in people with severe mental illness.^5,6^ Furthermore, trial data have estimated that antipsychotic treatment can substantially reduce violence, and observational data have found large reductions in violent criminality when substance misuse comorbidity is treated.^7,8^ Another relevant comorbidity to violent criminality is childhood conduct disorder.^9^

A public health approach to violence is underscored by the preventable nature of violence risk factors including victimisation, acute symptoms of mental illness, and comorbid substance misuse. A preventive approach needs liaison between multisector agencies, including criminal justice, substance misuse, and health care. Such an approach is also informed by secondary and tertiary prevention approaches in public health, in which high-risk groups are targeted as part of a national strategy. Consistent with this, higher rates of violence in people with severe mental illness, particularly in those untreated, than in individuals without mental illness have been reported: in a study in the UK, an estimated 14% of patients with first-episode psychosis studied were violent within 12 months,^10^ and a study in Sweden found a 20% increased risk of repeat violent offending in people with diagnosed schizophrenia-spectrum disorders released from prison.^11^ The wider national health-care context is relevant in this regard. In the UK, psychiatric inpatient bed numbers declined by 72% between 1987–88 and 2016–17,^12^ whereas mental health spending has been reduced from 2012 to 2016, with 40–50% of mental health trusts receiving budget reductions in cash terms.^13^ These trends are mirrored in the USA and other high-income countries.^14^

Economic studies can contribute to decisions about service provision by highlighting clinical areas with unmet needs and providing an estimate of the costs and potential savings of interventions. In mental health, cost-of-illness studies have highlighted the cost of specific outcomes, such as self-harm,^15^ and individual diagnoses.^16^ However, despite the importance of violence perpetration as a clinical outcome, its economic impact on society has rarely been assessed outside the area of substance misuse.^17^ The costs of violence are extensive, and the bearers of these costs are disparate, including victims, health services, and the criminal justice system. Consequently, it is necessary to adopt a broad scope when evaluating the costs associated with violence and assessing any preventive interventions. Many studies of the economic burden of schizophrenia and bipolar disorder do not include costs of violence or solely incorporate the costs to criminal justice.^16,18,19^ Economic evaluation in forensic mental health services, which manage violent psychiatric patients, has historically focused on highly selected populations and outcomes, although economic evaluations of personality disorder services for high-risk groups have been done.^20^

In this study, we aimed to provide a comprehensive estimate of the economic costs of violence perpetrated by people with severe mental illness and their distribution across sectors of the economy.

## Methods

### Study design

For this retrospective analysis, we developed a prevalence-based model to estimate the annual economic cost of violence perpetrated by individuals with severe mental illness in England and Wales between April, 2015, and March, 2016. We estimated the number and type of these violent incidents by using published data on severe mental illness prevalence, the association between severe mental illness and violent crime in epidemiological studies, the average number of crimes per perpetrator with severe mental illness, and the number of incidents of violence for the year 2015–16 in England and Wales (including violence not leading to conviction). The number of crimes were estimated separately for people with schizophrenia-spectrum disorders and bipolar disorders. Subsequently, the estimated number of crimes was multiplied by the unit cost to society per incident for each type of violence, reported by the UK Home Office, to estimate the economic impact by sector and type of violence. We then estimated the additional annual cost to the UK National Health Service (NHS) of attributable secure forensic mental health services. The model structure is presented in the appendix (p 2).

### Data selection and modelling procedures

To identify data to populate the model, MS searched PubMed and Google Scholar, complemented by reference screening and direct searching of grey literature through relevant organisations (Home Office, Office for National Statistics, Ministry of Justice, NHS England, and NHS Wales). In this process, secondary data from the UK were prioritised. Data and sources used in the model are described in the appendix (pp 5–8), along with additional information on model parameters (appendix pp 3–4).

The population of interest was comprised of individuals with severe mental illness, defined as schizophrenia-spectrum disorders and bipolar disorder. We identified the annual prevalence for schizophrenia-spectrum disorders (4·1 per 1000 individuals) from a meta-analysis of UK studies,^21^ and for bipolar disorder (7 per 1000) from worldwide estimates.^22^ These prevalence rates were applied to the Office for National Statistics mid-year population estimate for 2016 to estimate the population with severe mental illness.^23^

To quantify the association of severe mental illness with violence perpetration, we used risk estimates from longitudinal studies that were based on population-level registers. For schizophrenia-spectrum disorders, we identified a source that reported the ratio of odds ratios for violent crime between affected individuals and sibling controls.^5^ We converted this to a relative risk of 3·7 (95% CI 3·42–3·97) so that this input parameter was comparable to that for bipolar disorder. For bipolar disorder, we used a source reporting the ratio of relative risks of violent crime conviction of 3·5 (95% CI 2·98–3·99), comparing individuals with bipolar disorder with their unaffected siblings.^6^ Both data sources reported comparisons with sibling controls to account for sociodemographic and residual confounders, which is a more conservative approach than using matched population controls (where many confounders would not be accounted for, including familial factors such as early environment and shared genes). We selected these risk estimates from Swedish population studies because of the comprehensive, reliable, and valid coverage of the health and crime registers on which they were based and because of their longitudinal design (reducing the possibility of reverse causality). Generalisability from Swedish data to England and Wales is supported by consistent findings of similar rates of severe mental illness^24^ and violent assaults between these two countries;^25^ similar rates are also found among other northern European countries.

The relative risk for a violent conviction and prevalence of severe mental illness were then used to estimate the probability of a perpetrator of a violent incident having a severe mental illness. For the main analysis, we inputted the same relative risk of violence across all types of crime to provide a conservative estimate using comparisons with sibling controls. However, we explored this assumption in a sensitivity analysis.

We based our calculation of the absolute number of violent incidents on two official sources—the Crime Survey for England and Wales (CSEW) and Home Office data on the number of incidents by type of violent crime in 2015–16 (appendix pp 3, 5).^2,26^

Violence with a domestic perpetrator constitutes 20% of CSEW violence and is underreported in the face-to-face CSEW interview.^27^ This face-to-face interview was used to estimate the number of incidents of violence with and without injury. We inputted the difference in reporting between self-report questionnaires and face-to-face interview components of the CSEW as a factor to compensate for this underreporting (a 3·8-times increase in self-report questionnaires compared with that in face-to-face interviews).^27^

To account for recidivism, we used data on the average number of crimes per convicted perpetrator, which was 3·2 in people with severe mental illness and 2·3 in people without severe mental illness.^28^ Because the recividivism rate is higher in people with severe mental illness, the proportion of crimes perpetrated by those with severe mental illness is expected to be higher than the proportion of perpetrators (appendix p 3).

### Unit costs

We obtained the costs to society per crime for each type of violence for the year 2015–16 from a Home Office report,^2^ which grouped costs into the following three categories: annual costs of preventing and detecting crime; costs of lifetime physical and emotional harm to the victim from the crime, productivity loss in victim’s lifetime, NHS costs of treating victims immediately after crime, and annual costs of services provided by Victim Support (an independent charity partly funded by the Ministry of Justice); and costs in response to crime by the police and criminal justice system. These unit costs of crime were averaged across all violent incidents, including those not reported to police. Defensive spending refers to money spent on crime detection and prevention. Details of unit costs of crimes are presented in the appendix (p 6). The unit costs per crime include future costs of the incident (eg, long-term costs of harm to victims). Therefore, our estimated costs for 2015–16 represent the cost resulting from violent incidents occurring in that year, although these costs might be accrued in subsequent years.

A proportion of spending on secure mental health services, which provide care for psychiatric patients who pose a serious risk to others, can be attributed to violent crime in individuals with severe mental illness. We have estimated these costs on the basis of the NHS bed numbers and cost per bed per day for high, medium, and low secure services.^29,30^ These reference costs include the cost of private bed days funded by the NHS. Because not all patients in secure care have a severe mental illness as defined here, and some might not have committed violent offences, we estimated the proportion of patients with severe mental illness and history of violence perpetration within each level of secure services (appendix pp 3, 5).

### Sensitivity analyses

To address uncertainty, we did a probabilistic sensitivity analysis by drawing values from a-priori specified distributions of all model parameters simultaneously. In assigning distributions, we used reported SEs. When these were not reported, we assumed a relative SE of 20% (appendix p 4). Unit costs were incorporated deterministically. We did 10 000 iterations to obtain 95% CIs of the estimated costs.

Additionally, we did three deterministic sensitivity analyses to address key model assumptions. First, different estimates exist of the degree to which domestic violence is underreported in the face-to-face component of the Crime Survey for England and Wales.^27^ Therefore, the factor of underreporting of domestic violence was increased from 3·8 (main analysis) to 7·1 in a deterministic sensitivity analysis (appendix p 4).

Second, we used a different distribution of crime types for perpetrators with severe mental illness than for other perpetrators. We used different relative risks for the association of severe mental illness with arson and homicide, because evidence exists that these crimes are more strongly associated with severe mental illness than other types of crime.^31,32^ For the association between homicide perpetration and schizophrenia-spectrum disorders, we inputted an odds ratio (OR) of 19·5 (95% CI 14·7–25·8) from a meta-analysis of observational studies.^31^ For bipolar disorder, we did not identify any large-scale studies consistently reporting increased odds of homicide compared with those of other crime types, but noted that the prevalence of bipolar disorder among homicide offenders is much lower than that for schizophrenia-spectrum disorders.^33^ Therefore, we used the same association with homicide as for other crime types. For arson, we applied an OR for conviction of arson of 22·6 for men with schizophrenia-spectrum disorders, 38·7 for women with schizophrenia-spectrum disorders, 7·7 for men with bipolar disorder, and 27·5 for women with bipolar disorder. These associations were based on a case-control study of arson offenders using Swedish registers (appendix pp 4–5).^32^ The association between homicide, arson, and severe mental illness used in this sensitivity analysis is less conservative than that inputted in the main analysis, because the studies identified as data sources did not use a longitudinal design (so that the diagnosis could have occurred before or after the offence, although these are lifelong chronic disorders) and compared people with severe mental illness with controls from the general population rather than siblings.

We did a third deterministic sensitivity analysis, in which we adjusted the relative SE for input parameters (where this was not reported in the data source) from 20% to 30%.

For each deterministic sensitivity analysis, we repeated the probabilistic sensitivity analysis with 10 000 iterations of the model and illustrated the range of estimated costs using a tornado plot. We did all modelling and analysis in Microsoft Excel using Visual Basic for Applications.

### Role of the funding source

The funder of the study had no role in study design, data collection, data analysis, data interpretation, or writing of the report. All authors had full access to all the data in the study. The corresponding author had final responsibility for the decision to submit for publication.

## Results

Of a total of 4 507 500 violent incidents in 2015–16 in England and Wales, we estimated that 240 400 (5·3%, 95% CI 120 000–450 000) were committed by individuals with severe mental illness. This estimate comprised 31 homicides (<0·1%), 82 940 incidents of violence with injury (34·5%), 79 570 incidents of violence without injury (33·1%), 6490 rapes (2·7%), 60 660 other sexual offences (25·2%), 10 550 robberies (4·4%) and 178 cases of arson endangering life (<0·1%).

The total annual cost to society of violent crime perpetrated by individuals with severe mental illness in 2015–16 was estimated to be *£*2·5 (95% CI 1·4–4·5) billion (table), or 5·3% of a total estimated cost from violence of *£*47·1 billion for England and Wales. We examined the component parts of this cost according to type of violence, sector of spending, and diagnosis. Violence with injury led to the largest associated cost to society, followed by violence without injury, and sexual offences other than rape (table). The largest sector of spending contributing to these costs was lifetime physical and emotional harm to victims, followed by victims’ lost productivity (table).

**Figure 1:**
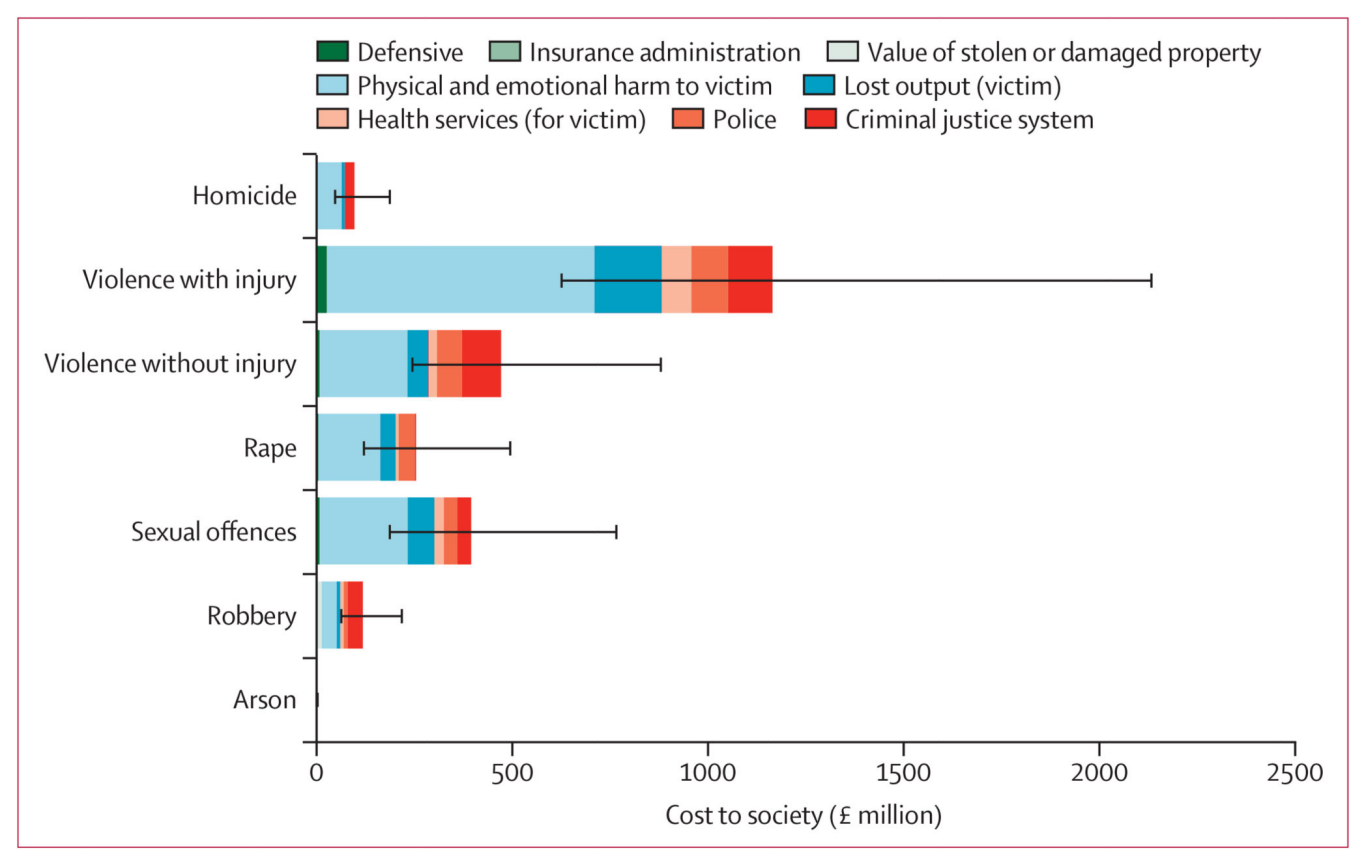
Overview of costs to society from violence perpetrated by people with severe mental illness, by type of violence and area of spending Error bars show 95% CIs for the total cost of each type of violence, derived from a probabilistic sensitivity analysis. Arson refers to incidents of arson endangering life.

The cost to health services from treating victims of violence occurring in 2015–16 was *£*136·7 (95% CI 74·3–246·3) million (table). The table and figure 1 show estimated total costs of violence perpetrated by individuals with severe mental illness by crime type and area of spending (costs separated by schizophrenia-spectrum and bipolar disorders are described in the appendix, p 9). The additional annual cost of secure forensic NHS services for individuals with severe mental illness and a history of violent crime was estimated to be *£*487·7 (95% CI 302·0–709·1) million, constituting *£*91·6 (55·6–127·5) million for high-secure care, *£*249·7 (94·8–445·5) million for medium-secure care, and *£*146·4 (59·0–257·6) million for low-secure care. We calculated the average annual cost per person with severe mental illness (on the basis of an estimated 648 000 individuals) to be *£*4630, including the costs of secure forensic services.

We did three sensitivity analyses that tested the assumptions in the main model. First, after increasing the factor of underreporting of domestic violence, the total cost of crime perpetrated by individuals with severe mental illness was increased to *£*3·0 (95% CI 1·6–5·6) billion. This increase was driven by the higher cost of domestic incidents of violence with and without injury, from *£*561·2 million to *£*1048·5 million (costs by crime type and sector are described in the appendix, p 10).

Second, after increasing the relative risk of homicide in individuals with schizophrenia-spectrum disorders, we estimated 43 homicides per year perpetrated by individuals with severe mental illness compared with 12 homicides in the main analysis. This increase resulted in higher costs due to homicide perpetrated by individuals with severe mental illness, from *£*98·7 (95% CI 45·6–190·7) million to *£*197·2 (109·7–312·7) million. Increasing the relative risk of arson perpetrated by individuals with severe mental illness resulted in an estimated 709 incidents of arson endangering life compared with 178 in the main analysis. This increased the cost of arson perpetrated by individuals with severe mental illness from *£*1·5 (95% CI 0·7–2·9) million to *£*6·0 (2·7–12·0) million. The total estimated cost of crime perpetrated by individuals with severe mental illness increased to *£*2·6 (95% CI 1·5–4·7) billion in this sensitivity analysis, or 5·6% of the total (appendix p 10).

**Figure 2:**
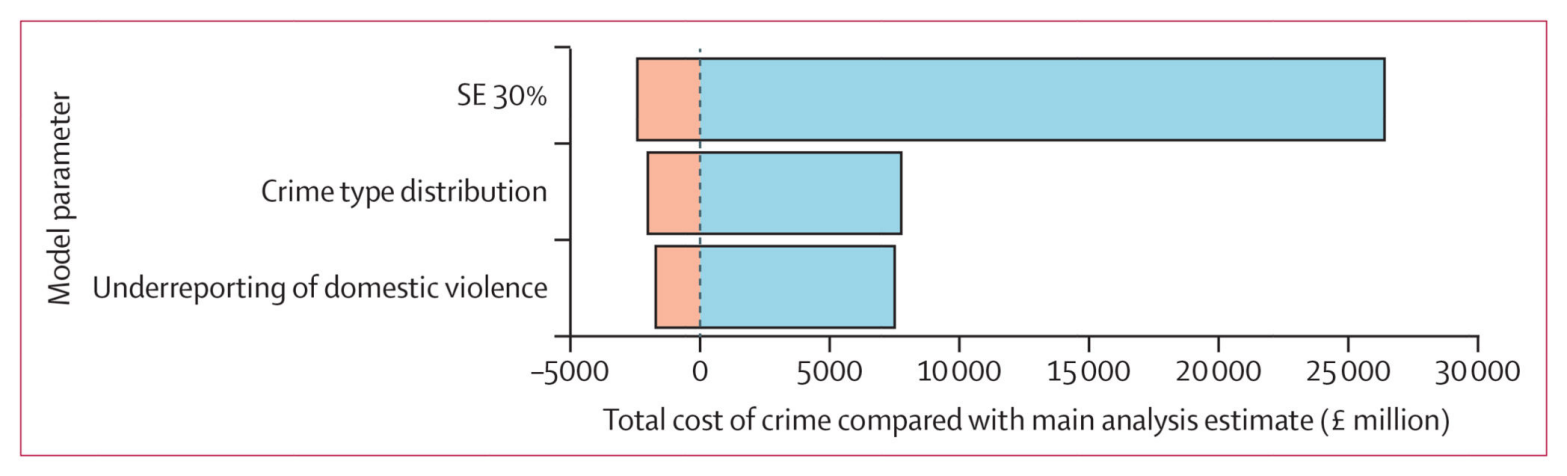
Impact of deterministic sensitivity analysis on model uncertainty Range of estimates for each deterministic sensitivity analysis, from 10 000 simulations. Values show variation from the deterministic point estimate. SE 30%: the assumed SE for the input parameter, when none was reported in the data source, was assigned at 30% of the mean (compared with 20% in the main analysis). Crime type distribution: relative risk of violence perpetrated by individuals with severe mental illness was varied according to crime type, with higher relative risk in homicide and arson than in other types of crime. Underreporting of domestic violence: the factor used to adjust for the underreporting of domestic violence was increased from 3·8 to 7·1.

Third, we assumed an SE of 30% of the mean for parameters where this was unavailable from data sources. This assumption did not change the deterministic estimate of the total cost from crime perpetrated by individuals with severe mental illness, but the degree of uncertainty (represented by 2·5th and 97·5th percentiles of cost estimates from 10 000 model iterations) changed from *£*1·4–4·5 billion to *£*0·9–6·4 billion. The effect of deterministic sensitivity analyses on model output uncertainty are shown in figure 2.

## Discussion

Our study estimated the total number of crimes perpetrated by individuals with severe mental illness in England and Wales in 2015–16 to be 240 400 (5·3% of all violent crimes), resulting in a total annual cost to society of *£*2·5 billion. The largest contributor to cost was physical and emotional harm to the victim, followed by lost productivity due to harms to victims. These estimates included the cost of violence that did not lead to criminal conviction and incorporated domestic violence. These findings represent a cost to society that has been overlooked in economic evaluations of severe mental illness. Moreover, they emphasise the importance of violence prevention from a novel perspective by highlighting the potential to reduce costs at a governmental and individual level. Overall, the proportionate costs of violent crime perpetrated by individuals with severe mental illness compared with the total violent crime cost is similar to estimates of population-attributable risks for violence.^28^ However, attributable risks have not accounted for the more conservative approach of using relative risks based on unaffected sibling controls or incident counts accounting for the underreporting of domestic violence. At the same time, the proportionate costs of violence perpetrated by people with severe mental illness could be considered small, at about 5% of the cost of all violent crimes.

Our findings have implications for economic evaluation of government spending on mental health and violence prevention because estimates of the economic impact of severe mental illness have rarely included costs from violence perpetration, yet our results suggest that this is a source of substantial cost to society.^18,19^ In the UK, the cost of schizophrenia and related conditions to society per patient was estimated to be *£*32 000 in one study and *£*66 000 in another (on the basis of 2016 prices).^34,35^ Despite their wide variation, neither estimate included comprehensive costs from violence perpetration. Where costs from violence perpetration were included, these were limited to prison-related ones. Our estimate would constitute 6–14% more than these previous calculations. Even in previous studies that included broader costs of crime, these costs were limited in scope. For example, one US investigation estimated the annual cost per patient with bipolar disorder to be *£*34 000, but included only costs of crime associated with substance misuse.^36^ Our findings show that these previous cost estimates overlooked substantial costs to society because they did not comprehensively incorporate violence perpetration. Therefore, inclusion of violence as a negative outcome might contribute meaningfully to future cost-of-illness studies and economic evaluations of severe mental illness.

Violence prevention has the potential to reduce costs in multiple areas of society, including in several publicly funded sectors. Our study adds a novel incentive to prioritise violence prevention in addition to other reported benefits for potential victims and perpetrators. Public health approaches have the potential to make a key contribution. However, the existing national strategy for violence prevention in England, for example, does not specifically consider violence prevention in the context of severe mental illness, although it does recognise the importance of drug use as a driver of violence.^37^ Our findings would underscore the importance of treating drug misuse, but suggest that this should be extended to alcohol misuse and also, specifically, to dual-diagnosis services for individuals with severe mental illness with these comorbidities. Access and provision of substance misuse services to people with severe mental illness is reportedly poorly resourced, subject to postcode variations, and mostly run by third-sector organisations.^38,39^ Furthermore, alcohol and drug treatment services are inadequately and inconsistently linked with mental health provision in most high-income countries and need considerable development.^38^ In the UK, national expert guidance has recognised that individuals with both substance misuse and mental illness are frequently excluded from health-care services, representing a substantial unmet need for these individuals.^39^ Our findings suggest that consideration of a long-term strategy and funding plan is warranted, which might involve more state-funded provision of health-care services and should recognise the potential for these services to reduce costs from violent offending by individuals with both substance misuse and severe mental illness. Research has shown that, of individuals with bipolar disorder convicted of a violent offence, about a quarter have a substance use disorder diagnosis,^6^ while half of individuals with schizophrenia convicted of violent offences have a diagnosis of comorbid substance use disorder.^31^ A conservative assumption is that comprehensive dual diagnosis services would reduce violent offending by individuals with comorbidity by 10%, which would lead to potential savings of *£*85 million when applied to our estimate.

Our findings also highlight an additional contribution of early intervention for severe mental illness, in which economic models do not consider the potential for first-episode psychosis services to contribute to reductions in crime and violence.^40,41^ Regarding repeat offending, we found that if the recidivism rate of individuals with severe mental illness was reduced to that of people without severe mental illness, the total annual cost to society from violent crime perpetrated by people with severe mental illness would be reduced from *£*2·5 to *£*1·8 billion, or a saving of *£*0·7 billion. This reduction underlines the importance of forensic and prison mental health services to decrease the impact of violence by reducing recidivism among the estimated 3200 prisoners with severe mental illness at any point in England and Wales.^42^ However, many perpetrators of violence who have severe mental illness do not come into contact with forensic or prison mental health services, particularly when violence is less severe or not reported to police or health professionals. Accordingly, interventions to reduce violence need to consider a broader public health approach and the involvement of more community and general mental health services.^43^ These interventions could target clinical features linked to violence, such as untreated psychosis and poor treatment adherence. ^43,44^

The strengths of our report include the high-quality epidemiological and cost data used in the analyses, much of which was based on population-level estimates. Additionally, we adopted a prevalence-based approach that overcomes the underreporting of violence perpetrated by individuals with severe mental illness, and the probabilistic sensitivity analysis enabled us to assess uncertainty surrounding the estimated costs by varying all input parameters simultaneously. The limitations of our study include generalisability to other countries where different patterns of violence and criminal justice responses exist, which might influence the cost per violent crime. However, the largest contributor to our estimate of economic impact was physical and emotional harm to the victim, calculated by use of a quality-adjusted life year approach. This cost might be more generalisable than costs to other sectors because it depends less on policing and criminal justice approaches. A second limitation was the assumptions underlying our models, which are explicit in the methods section and are primarily based on extrapolations from official sources of data and on generalisation from mainly Swedish population studies and meta-analyses from high-income countries. These assumptions will lead to uncertainty, particularly regarding the annual number of incidents of violent crime derived from the Crime Survey for England and Wales. Even within this large-scale survey, uncertainty is generated by scaling up incidents of events that are infrequent, or rarely reported, such as rape and other sexual offences. The degree of this uncertainty is not always reported and, therefore, we also made assumptions regarding the SE of data. However, the impact of this assumption on the findings was examined. Additionally, we have focused on violence perpetration by people with severe mental illness without considering victim information and thus, we have not estimated the additional costs to individuals who have severe mental illness who are also victims of violence. The latter is necessary to inform cost of illness studies for severe mental illness, but not directly relevant to our investigation that focused on perpetration.

Future cost-of-illness and cost-effectiveness studies of severe mental illness should consider including violence perpetration as an adverse outcome. Additionally, to more precisely account for the cost effect of domestic violence, which is prevalent and has a high economic burden,^45,46^ the epidemiology of its association with mental illness needs more clarification.

In conclusion, our study suggests that perpetration of violence by individuals with severe mental illness has economic impacts that should be considered in decision-making regarding the funding and cost-effectiveness of public health, mental health, and criminal justice initiatives to prevent violence perpetration. Our findings suggest that a preventive approach could reduce the economic cost to society, while improving the health of those with severe mental illness and improving public safety more broadly.

## Figures and Tables

**Table:** Total costs from violent incidents committed by individuals with severe mental illness, by area of spending and violence type

	Cost in anticipation		Cost as a consequence					Cost in response		Total (95% CI)
	Defensive	Insurance administration	Value of stolen or damaged property	Physical and emotional harm to victim	Lost output (victim)	Health services (victim)	Victim services	Police	Criminal justice system	
Homicide	1·9	0·0	0·0	63·9	7·8	0·03	0·2	0·4	24·6	98·7 (45·6–190·7)

Violence with injury	27·4	0·8	0·0	683·4	170·8	76·3	0·0	93·7	113·6	1166·1 (623·3–2135·0)

Violence without injury	8·8	0·8	0·0	223·6	53·3	21·5	0·8	64·5	99·5	472·6 (242·3–882·6)

Rape	6·3	0·07	0·0	158·4	38·3	7·2	0·3	41·3	3·8	255·6 (119·2–498·0)

Sexual offences other than rape	9·1	0·6	0·0	224·5	67·9	23·7	0·6	34·6	35·2	396·1 (185·1–768·5)

Robbery	2·0	1·5	10·9	37·9	9·7	8·0	0·1	10·7	38·7	119·4 (61·1–220·9)

Arson	0·02	0·04	0·3	0·2	0·06	0·03	0·002	0·2	0·7	1·5 (0·7–2·9)

Total (95% CI)	55·4 (30·2–99·7)	3·8 (2·1–6·9)	11·2 (5·7–20·6)	1391·7 (760·2–2503·2)	348·0 (190·0–628·8)	136·7 (74·3–246·3)	1·9 (1·0–3·5)	245·3 (133·6–441·3)	316·0 (171·1–571·9)	2510·1 (1370·5–4517·8)

Data are total cost (*£* million). 95% CIs show the 2·5th and the 97·5th percentile from probabilistic sensitivity analysis, with all input parameters varied simultaneously.

## Research in context

### Evidence before this study

We searched PubMed from inception to May 30, 2019, for articles in English, using the search terms (schizoph* OR “bipolar disorder” OR psychot*) AND (“economic burden” OR “cost of illness” OR “illness burden”). We identified a systematic review published in 2016 of cost-of-illness studies in schizophrenia and related conditions. Nine previous studies had included some legal costs related to violence, incorporating costs to criminal justice systems, but did not include costs to victims and other sectors of society. A 2013 systematic review of cost-of-illness estimates in bipolar disorder identified a single paper that addressed costs to the criminal justice system, whereas a 2018 cost-of-illness estimate in the USA incorporated some crime-related costs associated with substance misuse. In the UK, the Home Office published a report in 2018, estimating the cost to society per violent crime with a broad societal perspective, but did not stratify these costs according to mental health. The Home Office includes violent incidents not leading to conviction as examples of violent crime. These estimates have been used in economic evaluations for individuals in Dangerous and Severe Personality Disorder services in England and Wales, but we did not identify any studies of severe mental illness that incorporated these estimates.

### Added value of this study

To our knowledge, we provide the first comprehensive estimate of societal costs from violence perpetrated by individuals with severe mental illness. By incorporating official governmental estimates for unit costs of crime, we were able to examine which sectors of society bear the cost of violence, including physical and emotional harm to the victims, lost productivity for victims, and costs to health services and to the criminal justice system. Additionally, we were able to draw on recent, large-scale epidemiological data to estimate the association of severe mental illness with violence.

### Implications of all the available evidence

Our findings suggest that prevention of violence perpetration by individuals with severe mental illness might have substantial economic benefits, in addition to the well reported benefits for victims and perpetrators. Because the cost of violence perpetrated by people with severe mental illness is large relative to the total cost of illness, the assessment of violence should be included in future economic evaluation studies focused on severe mental illness. Violence prevention should focus on modifiable risk factors, such as substance misuse, and should involve public health approaches and coordinated efforts across forensic, prison, early intervention, and general adult mental health services.
